# Multi-omic diagnostics of prostate cancer in the presence of benign prostatic hyperplasia^☆^

**DOI:** 10.1016/j.heliyon.2023.e22604

**Published:** 2023-11-19

**Authors:** Matt Spick, Ammara Muazzam, Hardev Pandha, Agnieszka Michael, Lee A. Gethings, Christopher J. Hughes, Nyasha Munjoma, Robert S. Plumb, Ian D. Wilson, Anthony D. Whetton, Paul A. Townsend, Nophar Geifman

**Affiliations:** a School of Health Sciences, Faculty of Health and Medical Sciences, University of Surrey, Guildford, Surrey, GU2 7YH, United Kingdom; b The Hospital for Sick Children (SickKids), 555 University Ave, Toronto, ON M5G 1X8, Canada; c School of Biosciences, Faculty of Health and Medical Sciences, University of Surrey, Guildford, Surrey, GU2 7XH, United Kingdom; d Waters Corporation, Wilmslow, Cheshire, SK9 4AX, United Kingdom; e Manchester Institute of Biotechnology, Division of Infection, Immunity and Respiratory Medicine, Faculty of Biology, Medicine and Health, University of Manchester, Manchester, M13 9PL, United Kingdom; f Waters Corporation, Milford, MA 01757, USA; g Division of Systems Medicine, Department of Metabolism, Digestion and Reproduction, Imperial College, Burlington Danes Building, Du Cane Road, London, W12 0NN, United Kingdom; h Veterinary Health Innovation Engine, Faculty of Health and Medical Sciences, University of Surrey, Guildford, Surrey, GU2 7YH, United Kingdom; i School of Veterinary Medicine, Faculty of Health and Medical Sciences, University of Surrey, Guildford, Surrey, GU2 7YH, United Kingdom; j Division of Cancer Sciences, Manchester Cancer Research Center, Manchester Academic Health Sciences Center, University of Manchester, Manchester, M20 4GJ, United Kingdom

**Keywords:** Prostate cancer, Tumor progression, Biomarkers, LC-MS, Proteomics, Lipidomics, Complement, MAPK

## Abstract

There is an unmet need for improved diagnostic testing and risk prediction for cases of prostate cancer (PCa) to improve care and reduce overtreatment of indolent disease. Here we have analysed the serum proteome and lipidome of 262 study participants by liquid chromatography-mass spectrometry, including participants diagnosed with PCa, benign prostatic hyperplasia (BPH), or otherwise healthy volunteers, with the aim of improving biomarker specificity. Although a two-class machine learning model separated PCa from controls with sensitivity of 0.82 and specificity of 0.95, adding BPH resulted in a statistically significant decline in specificity for prostate cancer to 0.76, with half of BPH cases being misclassified by the model as PCa. A small number of biomarkers differentiating between BPH and prostate cancer were identified, including proteins in MAP Kinase pathways, as well as in lipids containing oleic acid; these may offer a route to greater specificity. These results highlight, however, that whilst there are opportunities for machine learning, these will only be achieved by use of appropriate training sets that include confounding comorbidities, especially when calculating the specificity of a test.

## 1 Introduction

Prostate cancer (PCa) is the fourth most common cancer globally in men [1,2]. Currently, Prostate-Specific Antigen (PSA) is commonly used as a diagnostic marker for PCa assessment, together with digital rectal examinations (DRE) and Magnetic Resonance Imaging (MRI). PSA levels can change with age, however, and so are stratified when used for diagnostic purposes; a 3 μg/L cut-off is used to identify potential PCa in men aged 50–59 years [3], with PSA values > 3 μg/L required for diagnoses of PCa in older men [4]. Elevated PSA can also be associated with non-neoplastic aetiology [5], leading to false positive PCa diagnoses [6]. Additionally, PCa cases have been reported where PSA levels were below 4 ng/mL, highlighting the risk of false negatives [7], and diagnosis is made more challenging by the high incidence of lower urinary tract symptoms (LUTS) in older men, especially where caused by benign prostatic hyperplasia (BPH). This potentially confounding condition can also be associated with elevated PSA [8], even though BPH is not a precursor to PCa [9], limiting the value of PSA as a biomarker.

Analysis of biofluids by mass spectrometry or NMR-based ‘omics techniques is a well-developed method in identifying markers for diagnosis and/or prognosis of various conditions, including PCa [7,10,11]. Recent studies have explored a variety of different multi-omics approaches to diagnosing PCa [12,13]; matrices including blood, urine, tissue and others have all been investigated in this way [14]. Such analyses frequently use machine-learning algorithms to process large high-dimensional datasets, but in many cases there is a risk that these analyses will introduce bias by training the models on idealised cohorts with clear distinctions between cases and healthy controls [15]. Such a focus, common in discovery studies, can generate tests with impressive sensitivity and specificity within a study setting, but with limited generalisability and applicability to patients in clinical settings [16]. The identification of biomarkers may be further complicated in the case of PCa by the existence of the blood-prostate barrier, which regulates the passage of substances between peripheral blood and the prostate tissue [[17], [18], [19]], potentially impeding the outward passage of disease-specific biomarkers into peripheral blood.

In this study, therefore, we aimed to identify biomarkers for PCa, and test these markers’ specificity with regard to BPH. Through this analysis, we aimed to utilise discovery LC-MS proteomics and lipidomics to help better understand how data-driven approaches can support clinical decision making when ground truths are uncertain and confounding comorbidities are present.

## 2 Materials and methods

### 2.1 Patients

The serum samples used in this work were provided by the SUN Biobank (NHS Ethics REC reference 18/YH/0314), selected from newly-diagnosed PCa patients (PCa, n = 127), patients with BPH (n = 37), and age-matched healthy controls (HC, n = 110) (Table 1). 12 participants had incomplete associated metadata and so were removed from the study (of which 1 was HC, 11 were PCa). HC participants presented with PSA levels below 1 ng/mL and a normal DRE. PCa patients were identified as previously described [10]; in brief, a positive PCa status was based on inclusion criteria of an abnormal prostate on DRE, or symptomatic patients with a high PSA level and abnormal biopsy, or a diagnosis made solely on the basis of a steep rise in PSA combined with LUTS. PCa patients were assessed as having neither metastasis nor nodal spread (tumours at Stages T1 through T3). Patients categorized as BPH presented with symptoms but did not meet the criteria for PCa as set out above. All participants recruited for this study provided written consent for the use of their samples and data; study approval was obtained from the Yorkshire & the Humber-Leeds East Research Ethics Committee (reference no. 08/H1306/115 + 5 and IRAS project ID 3582).

**Table 1:** Clinical summary data; patients were categorized as newly diagnosed with PCa (PCa), with BPH, or with neither (HC). PSA: Prostate Specific Antigen, BMI: Body Mass Index. Where specified ± determines standard deviation. Tumour progression based on TNM staging.

Group	Tumour Progression	Gleason Score	Age (Years)	PSA (ng/mL)	BMI
PCa – Newly Diagnosed	T1, n = 28	3 + 3(6) = 80	66.8 ± 7.4	12.1 ± 15.6	26.7 ± 3.5
(*n* = 116)	T2, n = 50	3 + 4(7) = 21			
	T3, n = 9	4 + 3 (7) = 5			
	Unknown n = 29	Other n = 10			
BPH	–	–	71.3 ± 8.9	5.9 ± 5.2	25.8 ± 4.2
(*n* = 37)					
Healthy Controls (HC)	–	–	64.8 ± 10.3	0.8 ± 0.7	26.9 ± 4.9
(*n* = 109)					
p-val (PCa vs HC)			0.032	<0.001	0.218
p-val (BPH vs HC)			<0.001	<0.001	0.099
p-val (PCa vs BPH)			0.017	0.003	0.068

### 2.2 Serum collection

Peripheral blood was collected into BD Vacutainer® red-capped collection tubes (BD Biosciences, USA). The samples were inverted five times and then incubated for 30 min at ambient room temperature, before being centrifuged (3000 rpm/1100 g, 10 min). The samples were processed within 2 h of collection and the serum (clear) fraction was stored at −80 °C until required for analysis. PSA levels were measured using the ADVIA Centaur system (Siemens, Ireland) [20].

### 2.3 Proteomics workflow

Sample preparation for the proteomics analysis used the procedure described by Muazzam [21], and was adopted for all calibrants, QCs, and samples. In short, sera samples were prepared by diluting 10 μL of serum with 46 μL of 0.1 % (w/v) RapiGest™ (Waters Corporation, Milford, MA) containing alcohol dehydrogenase (2 ng/μL) as internal standard in 50 mM ammonium bicarbonate. Samples were then incubated (80 °C, 45 min) to denature proteins; no proteins were removed. Following incubation, 100 mM DTT (11 μL) was added and incubated for a further 30 min at 60 °C to reduce the proteins, prior to alkylation with 200 mM iodoacetamide (3 μL) for 30 min at ambient room temperature. Trypsin 1:60 (w/w) (Gold Mass Spectrometry grade, Promega, Madison, WI, USA) was added to each sample for proteolytic digestion and the samples were then incubated overnight at 37 °C. Following incubation TFA was added to a final concentration of 0.5 % (v/v) to hydrolyse the RapiGest and the samples were heated (45 min at 37 °C) before centrifuging (25 min at 18,000 g). 30 μL of the supernatant was taken for LC-MS analysis. All samples were diluted prior to LC-MS analysis 1:4 (v/v) with 15 μL of 0.1 % FA (v/v).

Extracted peptides were separated using an ACQUITY™ Premier UPLC™ (Waters Corp., MA, USA) by reversed-phase chromatography. Peptides were injected (2 μL, 4 μg on-column) onto an ACQUITY Premier UPLC CSH™ C18 1.7 μm, 2.1 mm × 100 mm column (Waters Corp., MA, USA). The mobile phases were (A) water with 0.1 % (v/v) formic acid (A) and (B) acetonitrile with 0.1 % (v/v) formic acid. A 20 min gradient was employed for separation, consisting of 1–35 % mobile phase B over 16 min at a flow rate of 150 μL/min, maintaining the analytical column temperature at 55 °C. Lock mass consisting of [Glu1]-Fibrinopeptide with a flow rate of 5 μL/min was delivered to the mass spectrometry source.

Mass spectra were obtained using a SYNAPT™ XS mass spectrometer (Waters Corp., Wilmslow, UK) operated in positive electrospray ionisation (ESI) mode, with resolution set to 30,000 full-width half-maximum. The capillary voltage was 2.2 kV, cone voltage was 30 V and the source temperature was set at 100 °C. The scan time was set to 0.3 s. All data were acquired using Ultra-Definition MS^e^ to obtain fragmentation data simultaneously [[22], [23], [24]]; time-of-flight was externally calibrated over the acquisition mass range (50–2000 Da) before analysis with a NaCl mixture. Three technical replicates were acquired per sample; the order of samples and their replicates was randomised, and the spectra were collected using MassLynx v 4.2 software (Waters Corp., Wilmslow, UK).

Progenesis QI for Proteomics (Nonlinear Dynamics, Newcastle upon Tyne, UK) was used for retention time alignment, peak picking and normalization. Data were matched to an UniProt *Homo sapiens* database (release 2020_01) to provide protein identifications with a false discovery rate threshold (FDR) set to 1 %. A decoy database was generated as previously described [25]. Peptide and fragment ion tolerances were set automatically, allowing for one missed cleavage site. Cysteine carbamidomethylation was applied as a fixed modification to prevent the reactivity of cysteine residues with other molecules, whilst oxidation of methionines and deamidation of asparagine/glutamine were set as variable modifications.

### 2.4 Lipidomics workflow

Sample preparation for the lipidomics analysis used the procedure described by Sarafian et al. [26], and was adopted for all calibrants, QCs, and samples. In short, aliquots of plasma (25 μL) were transferred to low protein binding Eppendorf tubes followed by 125 μL of IPA/ACN (1:2, v/v) to precipitate proteins. Samples were vortex mixed for 1 min prior to incubation at −20 °C for 10 min. The samples were then shaken at 500 rpm at 5 °C for 2 h on a Thermo-Shaker PCMT (Grant-bio, Cambridge, 10.13039/100007472UK)to ensure complete protein precipitation. The extracted samples were then centrifuged at 10 300g for 10 min at 5 °C before the supernatant was transferred to total recovery glass vials (Waters, Milford, MA, USA) for LC-MS/MS analysis. Analysis was performed using an ACQUITY I-Class UPLC (Waters, Milford, MA, USA) following the method described by Munjoma et al.*;* [27] briefly, samples were loaded onto a 2.1 × 100 mm, 130 Å, 1.7 μm ACQUITY BEH™ Amide Column (Waters Corp., Milford, MA, USA) maintained at 45 °C using a flow rate of 0.6 mL/min. Mobile phase A was composed of 95 % ACN, 5 % 10 mM ammonium acetate (v/v), and mobile phase B was 50 % ACN, 50 % water, 10 mM ammonium acetate (v/v). The initial gradient conditions were 99.9 % mobile phase A, reducing to 80 % mobile phase A at 2 min and 20 % at 5 min before returning to initial conditions at 5.1 min. Initial conditions were held from 5.1 to 8 min, allowing for re-equilibration prior to the next injection. MS detection was performed on a Xevo™ TQ-XS mass spectrometer (Waters Corp., Wilmslow, UK) operating in multiple reaction monitoring ESI Mode. A range of authentic standards were used to create a library and optimize collision and cone voltages for the various lipid classes. A combination of LIPIDMAPS and the Human Metabolite Database were used to create theoretical fragments, which were also added to the library. Data were subsequently processed using a combination of TargetLynx™ (Waters Corp., Wilmslow, UK) and Skyline (MacCoss Lab, University of Washington, USA).

### 2.5 Data workflow

Data analysis and modelling was conducted in Python (3.8.10) using the Spyder Integrated Development Environment (5.2.2) [28]. 2.3 % of lipid and 8.9 % of protein observations were missing values; these were replaced with half-minimum values, following which the proteomic and lipidomic data sets were log-transformed to address heteroscedasticity. Data were unit-scaled and the combined dataset was initially inspected by principal component analysis to identify the presence or absence of major confounders [29]. The ability of the dataset to accurately describe the clinical groupings (i.e. cancer, BPH, and healthy controls) was then assessed by splitting the data into training and test sets (weighted for class balance). An XGB model implemented in Python using scikit-learn (Version 1.02) was trained and then tested on the populations of interest [30], with performance visualised by AUC, classification matrix and other metrics using the Yellowbrick library (Version 1.5) [31]. The XGB model was selected for its ability to cope with multicollinearity and robustness to the distributions and scaling of the feature set. Maximum depth for the model was set to 5, and all features were included in the initial model. Recursive feature elimination combined with leave-one-out cross validation was used on the training set to derive the biomarker panels with greatest diagnostic accuracy. All metrics were derived from the test set only, using a 60:40 train:test split and a random seed of 42. To compare statistical significance between the sensitivity and specificity of different tests (expressed as classification proportions), Pearson's Chi-squared test was used, with Yates' continuity correction employed to reduce Type 1 errors given the small incidence of some conditions in test sets. Confusion matrices were assessed by sensitivity, specificity, Youden's Index and Fisher's exact test.

### 2.6 Biomarker identification, annotation, and pathway analysis

Differentiating proteins were identified between the populations using ANOVA, via the MetaboAnalyst platform [32,33]. Pathway over-representation analysis using the proteomics data was performed using ClueGO (Version 2.5.7), a plug-in application in Cytoscape (Version 3.8.0) [34,35], searching against Reactome: Pathways and Reactome: Reactions (May 08, 2020). Proteomics pathway enrichment/depletion analysis was done using a two-sided hypergeometric test and with Bonferroni step down correction.

## 3 Results

### 3.1 Analysis of the serum proteome and lipidome to identify PCa diagnostic biomarkers

We analysed serum samples for 116 PCa newly diagnosed patients, with no previous cancer history (PCa or otherwise) or debilitating comorbidities, and where complete metadata were available, alongside 37 BPH patients. HCs (*n* = 109) had lower levels of PSA, did not have cancer or other known comorbidities, and were also younger than the BPH and PCa cohorts by an average of 6 and 3 years respectively (p-values of <0.001 and 0.03). Data on PSA levels, age, BMI and tumour stages are summarised in Table 1.

**Fig. 1:**
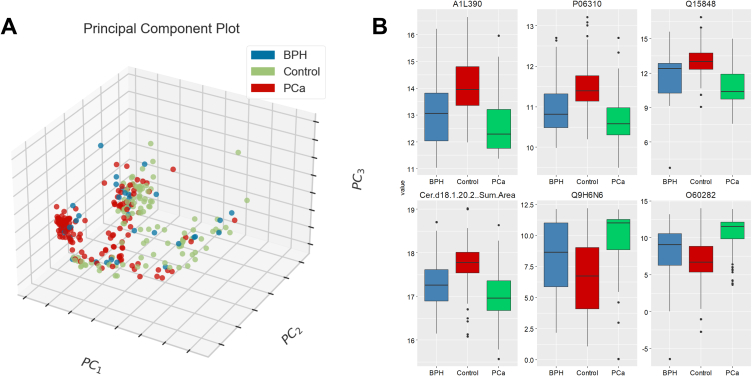
Overview of data structure: **(A)** PCA plot with first three components for PCa, BPH and HCs **(B)** Univariate boxplots of 6 statistically significant features measured by one-way ANOVA. Fisher's LSD post-hoc analysis is included in Table S1.

The proteomic and lipidomic analyses produced 1058 and 307 features respectively; Initial inspection by PCA showed limited unsupervised separation of the PCa/BPH/HCs classes (Fig. 1A); some clustering of PCa samples was evident (marked red in Fig. 1A) but not definitive. Analysis of the dataset by one-way ANOVA with Fisher's LSD post-hoc testing identified 758 features (out of the total of 1365) as statistically significant. The most highly ranked features by statistical significance were dominated by proteins rather than lipids. The 25 most differentiating features ranked by FDR p-value (calculated by one-way ANOVA) are set out in Table S1, Supplementary Material, including post-hoc analysis by Fishers LSD. Even where post-hoc analysis indicated statistical significance across all three pairings (Control - BPH; BPH - PCA; Control – PCA), however, BPH and PCa showed similarities in direction, especially in the six most significantly affected features (Fig. 1B).

**Fig. 2:**
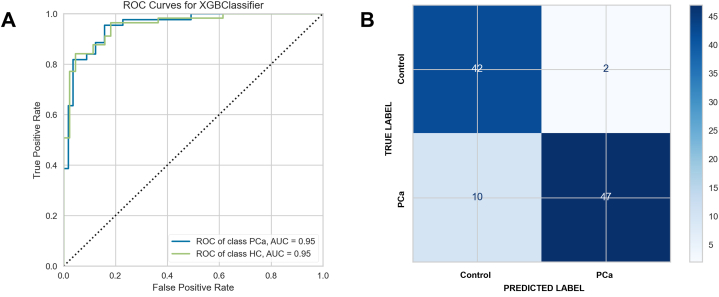
Ability of machine learning classifiers to identify participants by clinical diagnosis **(A)** test set AUC with ROC curves for PCa and HC participants **(B)** test set confusion matrix, PCa and HC participants, with Fisher's exact test p < 0.0001.

XGBoost classifier performance was good for the separation of PCa and healthy controls, with AUC for PCa and HCs of 0.95 and the confusion matrix showing good classification accuracy (Fig. 2A and B). The 20 most differentiating features ranked by FDR p-value (calculated by Student t-test) are set out in Table S2, Supplementary Material.

Where classifiers overlap, sensitivity and specificity may be more useful measures [36]. These metrics plus Youden's Index together with their 95 % confidence intervals are shown in Table 2, derived from the confusion matrix in Fig. 2B.

**Table 2:** Diagnostic metrics for PCa versus HCs including confidence intervals.

Group: 2-class problem	Sensitivity	Specificity	Youden's Index
Healthy Controls	0.95 (0.85–0.99)	0.82 (0.70–0.91)	0.78 (0.55–0.91)
Prostate Cancer	0.82 (0.70, 0.91)	0.95 (0.85–0.99)	0.76 (0.55–0.91)

**Fig. 3:**
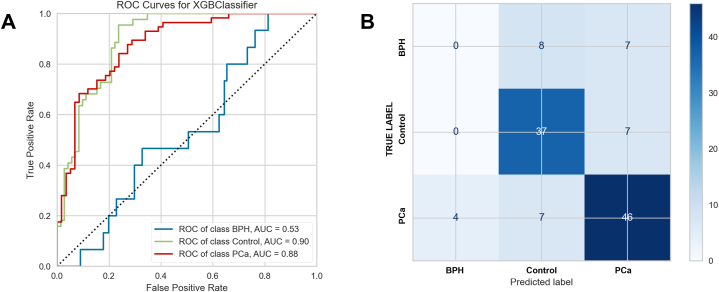
Ability of machine learning classifiers to identify participants by clinical diagnosis (A) test set AUC with ROC curves for HC, BPH and PCa participants (B) test set confusion matrix, HC, BPH and PCa participants, with Fisher's exact test p < 0.0001.

An XGBoost classifier was then trained and tested on the data set inclusive of PCa, HC and BPH classes. The machine learning classifier performance was reasonable for the separation of PCa and healthy controls, with AUC for PCa of 0.86 and for HCs of 0.91 (Fig. 3A). The model performed less well though for the identification of BPH, with AUC of 0.48. BPH was misclassified equally as PCa and HC (Fig. 3B).

Sensitivity, specificity and Youden's Index with their 95 % confidence intervals are shown in Table 3, calculated as ‘one class versus all’. For PCa, sensitivity declined modestly from 0.82 to 0.76, and specificity declined from 0.95 to 0.77. The decline in sensitivity (the proportion of PCa cases correctly classified) was not significant at the 95 % level, with a p-value of 0.641. The decline in specificity was significant at the 95 % level, with a p-value of 0.017.

**Table 3:** Diagnostic metrics for three classes including confidence intervals.

Group: 3-class problem	Sensitivity	Specificity	Youden's Index
Healthy Controls	0.71 (0.56–0.84)	0.83 (0.72, 0.91)	0.54 (0.28–0.75)
BPH	0.15 (0.02–0.45)	0.87 (0.79–0.93)	0.03 (-0.19–0.39)
Prostate Cancer	0.76 (0.63–0.86)	0.77 (0.64–0.87)	0.54 (0.28–0.74)

For completeness, an XGBoost model was trained for the two-class problem (BPH versus HC) which generated AUC of 0.62 for BPH, improved versus the 0.48 in the three-class problem (Table S3 and Fig. S1, Supplementary Material), but with material overlap in the differentiating features between PCa versus HCs and BPH versus HCs.

### 3.2 Pathway analysis of BPH and PCa

For PCa versus HC, over-representation analysis produced a list of statistically significant pathways which had been dysregulated. The full list is presented in Table S4, Supplementary Material; the 10 with greatest statistical significance are summarised in Table 4.

**Table 4:** Pathway analysis using protein biomarkers separating PCa from HC: pathways shown by number of genes identified. Pathways with 4+ associated genes and statistical significance (p-value <0.05) are shown; p-values are corrected by Bonferroni step-down.

Term	Group FDR p-value	% Associated Genes
Regulation of Complement cascade	4.99E-20	46.8
Activation of C3 and C5	4.99E-20	75.0
Initial triggering of complement	4.99E-20	43.5
Complement cascade	4.99E-20	46.6
Response to elevated platelet cytosolic Ca2+	4.00E-13	24.6
Platelet activation, signaling and aggregation	4.00E-13	16.0
Platelet degranulation	4.00E-13	25.6
Hemostasis	4.00E-13	11.9
Formation of Fibrin Clot (Clotting Cascade)	3.82E-12	43.6
Common Pathway of Fibrin Clot Formation	3.82E-12	54.5

As a second step, BPH versus HC was analysed in the same way. Pathway analysis produced a list of statistically significant pathways which had been dysregulated. The full list is presented in Table S5, Supplementary Material; the 10 with greatest statistical significance are summarised in Table 5. All of the pathways identified as differentiated between BPH and HCs were also differentiated between PCa and HCs.

**Table 5:** Pathway analysis using protein biomarkers separating BPH from HC: pathways shown by number of genes identified. Pathways with 4+ associated genes and statistical significance (p-value <0.05) are shown; p-values are corrected by Bonferroni step-down.

Term	Group FDR p-value	% Associated Genes
Hemostasis	2.09E-06	4.8
Factors involved in megakaryocyte development and platelet production	1.88E-04	6.5
Intrinsic Pathway of Fibrin Clot Formation	1.12E-04	26.1
Formation of Fibrin Clot (Clotting Cascade)	1.12E-04	15.4
Regulation of Insulin-like Growth Factor (IGF) transport and uptake by Insulin-like Growth Factor Binding Proteins (IGFBPs)	3.74E-05	8.8
Post-translational protein phosphorylation	3.74E-05	9.3
Complement cascade	1.70E-09	20.7
Regulation of Complement cascade	1.70E-09	23.4
Platelet degranulation	1.58E-05	10.1
Platelet activation, signaling and aggregation	1.58E-05	6.5

### 3.3 Supervised separation of BPH and PCa

**Fig. 4:**
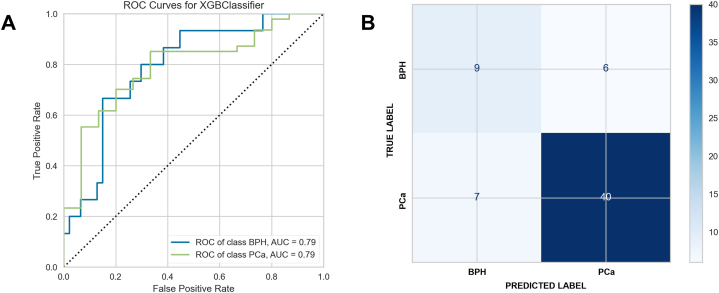
Ability of machine learning classifiers to identify BPH versus PCa **(A)** test set AUC with ROC curves for BPH versus PCa **(B)** test set confusion matrix, BPH versus PCa, with Fisher's exact test p = 0.001.

A model was then constructed to separate BPH from PCa, i.e. the two conditions in isolation. AUCs were higher for BPH with the model focused on separating only two classes (Fig. 4A), with improved classification accuracy (Fig. 4B). The AUCs improved using this test as a stratified diagnostic, albeit this is an idealised model that benefits from the removal of all HCs, so is not directly comparable with the results shown in Fig. 3.

Key metrics are shown in Table 6, and the 50 most differentiating features are summarised in Table S6, Supplementary Material.

**Table 6:** Diagnostic metrics for BPH versus PCa including confidence intervals.

Group: 2-class problem	Sensitivity	Specificity	Youden's Index
BPH	0.60 (0.32–0.84)	0.85 (0.72, 0.94)	0.45 (0.04–0.77)
Prostate Cancer	0.85 (0.72–0.94)	0.60 (0.32, 0.84)	0.45 (0.04–0.77)

A smaller number of pathways differentiated BPH and PCa when compared with the two conditions separately contrasted with HCs. These are set out in Table 7. The complement cascade again featured. Platelet activation and the Ras/Raf/Map Kinase pathway were the main other pathway categories of pathways affected. The latter is employed for agonist stimulated activation of platelets, often involving lipid mediators. The Ras/Raf/MAP Kinase pathways were featured in the full list differentiating PCa versus HC (Table S5, Supplementary Materials), but not in the full list differentiating BPH versus HC (Table S4 Supplementary Materials), consistent with this being a specific area of difference between the two conditions.

**Table 7:** Pathway analysis using protein biomarkers segregating BPH from PCa: pathways shown by number of genes identified. Pathways with 4+ associated genes and statistical significance (p-value <0.05) are shown; p-values are corrected by Bonferroni step-down.

Term	Group FDR p-value	% Associated Genes
Complement cascade	1.06E-13	22.4
Regulation of Complement cascade	1.06E-13	23.4
Common Pathway of Fibrin Clot Formation	2.95E-06	27.3
Formation of Fibrin Clot (Clotting Cascade)	2.95E-06	20.5
Platelet degranulation	3.81E-06	10.1
Post-translational protein phosphorylation	2.24E-05	10.2
MAP2K and MAPK activation	3.87E-04	15.0
Signaling by moderate kinase activity BRAF mutants	3.87E-04	12.8
Signaling by high-kinase activity BRAF mutants	3.87E-04	16.7
Signaling by RAS mutants	3.87E-04	12.8
Signaling by BRAF and RAF1 fusions	3.87E-04	10.4
Paradoxical activation of RAF signaling by kinase inactive BRAF	3.87E-04	12.8
Platelet Aggregation (Plug Formation)	3.87E-04	12.8
Signaling downstream of RAS mutants	3.87E-04	12.8
Signaling by RAF1 mutants	3.87E-04	11.6
Initial triggering of complement	2.76E-03	17.4
Plasma lipoprotein remodeling	3.80E-03	12.1
NR1H3 & NR1H2 regulate gene expression linked to cholesterol transport and efflux	4.23E-03	10.8
Interaction between L1 and Ankyrins	4.38E-03	12.9

Although not incorporated within pathways analysis, a ceramide (d18:1/18:2), diglyceride (18:1/18:2) and monoglyceride (18:1) were represented in the top 20 features identified by the XGBoost classifier. These entities were not platelet activators *per se*.

## 4 Discussion

In this work, isolating PCa and HC participants in an idealised case-control two-class cohort identified biomarkers with good sensitivity (0.82), specificity (0.95) and AUC (0.95) for the diagnosis of PCa. The introduction of a ‘real-world’ confounding class, however, caused a deterioration in these metrics. For a three-class model that included BPH, AUC was 0.86 for classification based on PCa, 0.91 for healthy controls, and just 0.48 for BPH, with the algorithm classifying 47 % of BPH participants as belonging to the PCa class. This led to specificity for PCa falling from 0.95 to 0.77, a statistically significant decline (p-value 0.017 by Pearson's chi-square). These results are strongly suggestive of powerful confounders in the dataset, with BPH related dysregulation showing changes in many of the same individual proteins/lipids (Fig. 1) and pathways (Table 4, Table 5) as PCa. Given that over half of men aged 50+ will have BPH, which may also be coincident with PCa [37], or in combination with other LUTS, we hypothesis that there will be considerable difficulty in establishing diagnostic ground truths. This is an example of the poor generalisability of machine learning algorithms when trained on two-class problems.

Analysis of biomarkers and pathways separating PCa and HCs shows the complement cascade as heavily implicated. This is consistent with literature descriptions of the complement system being associated with prostate cancer; previous reports concentrating on a two-class model have identified proteins in the complement cascade as markers for prostate cancer with robust AUCs [10,38]. The fact that similar pathways were dysregulated in BPH, however, is a cause for caution around the specificity of proteins from the complement cascade. The complement cascade has been associated with cancers more broadly, as a source of powerful proinflammatory molecules such as C3a and C5a [39]. These complement anaphylatoxins can drive chronic inflammation as well as inducing angiogenesis [40], therefore encouraging the genesis and metastatic spread of neoplastic cells [[41], [42], [43]], but the complexity of complement pathways and number of associated proteins hinders the identification of simple causal relationships [44,45]. In this work we also associate the complement cascade to BPH, in line with its characterisation as an immune inflammatory disease [46,47]. This finding also highlights the potential for pathways related to poor health and generalised inflammation to be mis-identified as disease specific. UK Biobank metabolomics data have shown a similar phenomenon of common biomarkers being associated with different disease conditions [48].

To investigate whether any biomarkers might be able to separate BPH from PCa, an additional model was constructed. This performed better, with AUC for BPH of 0.79, albeit with the benefit of HCs being excluded. Pathway analysis of BPH versus PCa produced some pathways common to all conditions (complement activation and regulation, for example). This is suggestive that these pathways are altered to different degrees between the two conditions, but with enough overlap to prevent accurate separation. Pathways unique to the BPH versus PCa model were dominated by platelet activation and the related molecular signaling pathway that triggers this event, Ras/Raf/mitogen-activated protein kinase (MAPK) cascades. These MAP Kinase-related pathways included the upstream elements of Ras and Raf, both mutated in human cancers, resulting in dysregulated signaling and promoting cell proliferation and tumour development [49]. A relationship between BRAF mutations and lipid metabolites has previously been associated to tumor progression [50]; also relevant is the concept of tumour-induced platelet activation [51,52]. Prostate cancer cells interact with platelets directly and are sensitive to this interaction as exemplified by increased cell survival and invasive properties [53].

In addition to the pathway analysis, several lipids were also identified as differentiating BPH from PCa, all of which contained the fatty acid oleic acid (18:1). Stearic to oleic acid ratios and stearic acid concentrations have previously been found to be increased in adipose tissue for patients with BPH relative to PCa, and oleic acid has been associated with the proliferation of malignant prostate cancer cells [54], albeit potentially only in the presence of specific fatty acid binding proteins [55]. Modulation of fatty acid composition has the potential to alter membrane composition and hence fluidity. It should, however, be recognised that oleic acid is the most common fatty acid in human metabolism, and no definitive link has been identified; thus caution should be exercised in over-interpreting these results. Nonetheless, in situations where a definitive diagnosis of prostatic disorder has been made, a biomarker panel including features such as oleic acid or MAPK may have the potential to improve the specificity of PCa blood testing, so assisting clinical decision making. This is especially relevant given the shortfalls in timely and specific diagnostic blood tests currently in use, for example PSA-based tests [[5], [6], [7]]. These markers therefore represent useful targets for future investigation, for example by quantitative targeted LC-MS analyses.

These results also highlight a widespread issue in machine learning applied to ‘omics data: over-statement of specificity by confusing general biomarkers for poor health or inflammation with specific biomarkers for a condition, an increased risk when machine learning models are trained on idealised case-control discovery studies. Whilst reasons of cost or practicality will mean that two-class cohorts can still be worthwhile in specific use-cases, such studies by definition cannot test specificity of identified biomarkers with regard to closely-related confounders and can produce biased estimates of specificity and other metrics.

We would highlight a number of limitations in this work. The groupings (BPH, HC, PCa) were not age matched, as set out in Table 1, which could represent a confounding factor, and the size of the population was limited with *n* of 262, and especially with *n* of 37 in the group clinically diagnosed as presenting with BPH. Reweighting algorithms can compensate for this but are a second-best solution. In addition, the existence of the blood-prostate barrier potentially limits peripheral blood as a sampling matrix in this particular use-case. These limitations could be addressed in a larger prospective study, and/or by investigating sampling matrices other than peripheral blood. Furthermore, whilst information on participants was collected including medication regimes and family history of PCa, comprehensive medical histories were not obtained, preventing analysis of the full range of possible confounders.

Nonetheless, in this work we demonstrate that patients with PCa can be separated from HCs, but that many of the pathways responsible for this separation are not specific to PCa. We also show that biomarkers associated with MAP Kinase and oleic acid may show promise in separating BPH from PCa, but as yet with insufficient accuracy to meet clinical requirements. Further work to investigate these markers could have the potential to increase the specificity of PCa diagnostic tests. More broadly, given closely related confounders such as BPH, future work in this area should broaden its horizons beyond idealised two-class cohorts, both for proteomic and multi-omic studies. Given the widespread prevalence of BPH and other LUTS, this will be essential in order to avoid training machine learning algorithms on datasets that do not accurately represent the clinical reality of uncertain ground truths and overlapping conditions.

Supplementary Materials: The following supporting information is included. Table S1: p-values for the 25 most differentiating features between HC, BPH and PCa participants, ranked by FDR p-value using ANOVA. Table S2: fold-changes and p-values for the 50 most differentiating features between PCa and HC participants, ranked by FDR p-value. Table S3: fold-changes and p-values for the 50 most differentiating features between BPH and HC participants, ranked by FDR p-value. Fig. S1: ability of machine learning classifiers to identify participants by clinical diagnosis (A) test set AUC with ROC curves for HC and BPH participants (B) test set confusion matrix, HC and BPH participants. Table S4: pathway analysis using protein biomarkers separating PCa from HC: pathways shown by number of genes identified. Pathways with at least 4 protein biomarkers and with statistical significance (p-value <0.05) are shown; p-values are corrected by Bonferroni step-down. Table S5: pathway analysis using protein biomarkers separating BPH from HC: pathways shown by number of genes identified. Pathways with a minimum of 4 protein biomarkers and with statistical significance (p-value <0.05) are shown; p-values are corrected by Bonferroni step-down.

## Funding

Funding for this work was provided by Punjab Educational Endowment Fund (PEEF) to P.A.T. to support A.M. The work was also supported by 10.13039/501100007903Bloodwise (Award 19007) to A.D.W. P.T. was additionally supported by the Hope for Guernsey and Male Uprising in Guernsey charities.

**Institutional Review Board Statement**: This study was carried out following the rules of the Declaration of Helsinki of 1975, revised in 2013. Study approval was obtained from the Yorkshire and the Humber-Leeds East Research Ethics Committee (reference no. 08/H1306/115 + 5 and IRAS project ID 3582).

**Informed Consent Statement**: Informed consent was obtained from all subjects involved in the study.

## Data Availability statement

In addition to the data presented in the paper or in Supplementary Information, proteomic data for this study can be accessed via ProteomeXchange with the identifier PXD046530 and lipidomic data can be accessed via EMBL-EBI MetaboLights with the identifier MTBLS3272.

## Funding

Funding for this work was provided by Punjab Educational Endowment Fund; support was also received from 10.13039/501100007903Bloodwise (Award 19007) as well as from the Hope for Guernsey and Male Uprising in Guernsey charities.

## CRediT authorship contribution statement

**Matt Spick:** Writing – original draft, Visualization, Validation, Software, Investigation, Formal analysis, Data curation. **Ammara Muazzam:** Methodology, Investigation, Data curation. **Hardev Pandha:** Resources. **Agnieszka Michael:** Resources, Investigation. **Lee A. Gethings:** Writing – review & editing, Methodology, Investigation, Data curation. **Christopher J. Hughes:** Methodology. **Nyasha Munjoma:** Methodology. **Robert S. Plumb:** Methodology. **Ian D. Wilson:** Writing – review & editing, Resources, Methodology. **Anthony D. Whetton:** Writing – review & editing, Supervision, Project administration, Funding acquisition, Conceptualization. **Paul A. Townsend:** Writing – review & editing, Supervision, Resources, Conceptualization. **Nophar Geifman:** Writing – review & editing, Supervision, Project administration, Conceptualization.

## Declaration of competing interest

The authors declare the following financial interests/personal relationships which may be considered as potential competing interests.
