# Supplementary material for: Multi-omic diagnostics of prostate cancer in the presence of benign prostatic hyperplasia
Source: Heliyon. 2023 Nov 19;9(12):e22604. doi: 10.1016/j.heliyon.2023.e22604 (PMC10709398; doi:10.1016/j.heliyon.2023.e22604)
Supplement: Multimedia component 1 [file mmc1.docx]

Multi-omic diagnostics of prostate cancer in the presence of confounding conditions and uncertain ground truths: Supplementary Materials

**Table S1**: P-values for the 25 most differentiating features between HC, BPH and PCa participants, ranked by FDR p-value using ANOVA

| **Feature** | **f.value** | **p.value** | **FDR p.value** | **Fisher's LSD** |
| --- | --- | --- | --- | --- |
| Q15848 | 59.51 | 5.44E-22 | 7.42E-19 | Control - BPH; BPH - PCA; Control - PCA |
| Cer(d18:1/20:2) Sum Area | 55.93 | 6.46E-21 | 4.40E-18 | Control - BPH; BPH - PCA; Control - PCA |
| O15078;A1L390;P61011;Q96FX2;Q96QE5;Q96T23;Q9BQ75;Q9Y2R4 | 55.02 | 1.22E-20 | 5.53E-18 | Control - BPH; BPH - PCA; Control - PCA |
| Q6ZWK4 | 52.97 | 5.18E-20 | 1.76E-17 | BPH - Control; PCA - BPH; PCA - Control |
| A0MRZ7 | 50.76 | 2.52E-19 | 6.87E-17 | Control - BPH; BPH - PCA; Control - PCA |
| P06310;75B6S6 | 50.15 | 3.91E-19 | 8.87E-17 | Control - BPH; BPH - PCA; Control - PCA |
| 75B6S2;A2NJV5 | 49.55 | 6.02E-19 | 1.17E-16 | Control - BPH; BPH - PCA; Control - PCA |
| Q5JSL3 | 46.21 | 6.91E-18 | 1.18E-15 | Control - BPH; BPH - PCA; Control - PCA |
| 75B6P5;P01615 | 45.78 | 9.46E-18 | 1.29E-15 | Control - BPH; BPH - PCA; Control - PCA |
| 87WW87;P01614 | 45.78 | 9.46E-18 | 1.29E-15 | Control - BPH; BPH - PCA; Control - PCA |
| Q9Y618 | 45.48 | 1.18E-17 | 1.47E-15 | BPH - Control; PCA - BPH; PCA - Control |
| P08123 | 43.77 | 4.21E-17 | 4.78E-15 | BPH - Control; PCA - BPH; PCA - Control |
| P01042 | 43.64 | 4.63E-17 | 4.85E-15 | Control - BPH; BPH - PCA; Control - PCA |
| Q96NN9 | 42.58 | 1.02E-16 | 9.97E-15 | Control - BPH; BPH - PCA; Control - PCA |
| P04217 | 41.82 | 1.83E-16 | 1.66E-14 | Control - BPH; BPH - PCA; Control - PCA |
| Q9H6N6 | 41.60 | 2.15E-16 | 1.83E-14 | BPH - Control; PCA - BPH; PCA - Control |
| P07357 | 39.70 | 9.13E-16 | 7.32E-14 | Control - BPH; BPH - PCA; Control - PCA |
| Q8TD84 | 39.46 | 1.10E-15 | 8.32E-14 | Control - BPH; BPH - PCA; Control - PCA |
| O60282 | 38.54 | 2.23E-15 | 1.60E-13 | BPH - Control; PCA - BPH; PCA - Control |
| J9YXX1 | 38.16 | 3.00E-15 | 2.04E-13 | Control - BPH; BPH - PCA; Control - PCA |
| P02652 | 37.72 | 4.21E-15 | 2.73E-13 | Control - BPH; BPH - PCA; Control - PCA |
| Q9H668 | 36.58 | 1.02E-14 | 6.31E-13 | BPH - Control; PCA - BPH; PCA - Control |
| B4J1X5 | 36.46 | 1.12E-14 | 6.40E-13 | Control - BPH; BPH - PCA; Control - PCA |
| Q15772 | 36.45 | 1.13E-14 | 6.40E-13 | BPH - Control; PCA - Control |

**Table S2**: Fold-changes and p-values for the 50 most differentiating features between PCa and HC participants, ranked by FDR p-value

| **Feature** | **FDR p-value** | **Log2FoldChange** | **Feature** | **FDR p-value** | **Log2FoldChange** |
| --- | --- | --- | --- | --- | --- |
| Q15848 | 2.78E-23 | 0.26 | Q6GTS8 | 7.11E-14 | 0.05 |
| O15078;A1L390;P61011;Q96FX2;Q96QE5;Q96T23;Q9BQ75;Q9Y2R4 | 4.62E-20 | 0.16 | Q9NTW7 | 8.61E-14 | -2.21 |
| Cer(d18:1/20:2) Sum Area | 2.15E-19 | 0.06 | O75051 | 8.66E-14 | 0.17 |
| Q6ZWK4 | 4.68E-19 | -3.72 | Q96KH6 | 9.58E-14 | -2.53 |
| A0MRZ7 | 1.43E-18 | 0.14 | Q6ZMZ3 | 1.01E-13 | -0.47 |
| P06310;75B6S6 | 2.34E-18 | 0.10 | P19827 | 1.03E-13 | 0.04 |
| 75B6S2;A2NJV5 | 3.12E-18 | 0.13 | Q5TZA2;Q12882 | 1.37E-13 | 0.20 |
| 75B6P5;P01615 | 2.31E-17 | 0.20 | P49454 | 1.71E-13 | -0.19 |
| 87WW87;P01614 | 2.31E-17 | 0.10 | P07996 | 1.71E-13 | 0.14 |
| P01042 | 4.37E-17 | 0.06 | Q9C0D2 | 2.02E-13 | 0.16 |
| Q5JSL3 | 5.18E-17 | 0.16 | Q9H223 | 2.65E-13 | -1.16 |
| Q9Y618 | 6.87E-17 | -2.07 | Q9P2D1 | 2.85E-13 | 0.05 |
| P04217 | 1.20E-16 | 0.05 | Q8WYA0 | 6.31E-13 | 0.60 |
| P08123 | 1.92E-16 | -2.29 | P36980 | 9.53E-13 | 0.23 |
| Q96NN9 | 2.10E-16 | 0.08 | Q86YW9 | 1.08E-12 | -0.29 |
| O60282 | 8.26E-16 | -0.56 | Q9H0R5 | 1.08E-12 | -2.29 |
| Q9H6N6 | 9.66E-16 | -0.57 | P35453 | 1.12E-12 | -0.75 |
| P07357 | 1.37E-15 | 0.57 | Q9BWT6 | 1.14E-12 | 0.17 |
| Q8TD84 | 1.84E-15 | 0.08 | Q9BX63 | 1.32E-12 | 0.44 |
| B4J1X5 | 5.38E-15 | 0.28 | A0AVK6 | 1.63E-12 | 0.06 |
| J9YXX1 | 8.11E-15 | 0.39 | Q96L33 | 1.81E-12 | -2.50 |
| Q14185 | 8.27E-15 | 0.16 | P01714 | 1.85E-12 | 0.24 |
| P02652 | 1.51E-14 | 0.07 | Q8N1T3;Q9Y314 | 3.50E-12 | 0.07 |
| Q9H668 | 3.26E-14 | -1.83 | Q9BXT8 | 3.53E-12 | -0.30 |
| Q15772 | 5.44E-14 | -1.43 | P10643 | 3.96E-12 | 0.05 |

**Table S3**: Fold-changes and p-values for the 50 most differentiating features between BPH and HC participants, ranked by FDR p-value

| Feature | FDR p-value | Log2FoldChange | Feature | FDR p-value | Log2FoldChange |
| --- | --- | --- | --- | --- | --- |
| Q9UIF7 | 9.22E-03 | 6.86 | Q96L33 | 1.23E-02 | 1.81 |
| Q6ZWK4 | 9.22E-03 | 2.77 | P03952 | 1.31E-02 | -0.06 |
| P06310;75B6S6 | 9.22E-03 | -0.07 | Q06033 | 1.35E-02 | -0.13 |
| 75B6S2;A2NJV5 | 9.22E-03 | -0.08 | P02652 | 1.35E-02 | -0.04 |
| A0MRZ7 | 9.22E-03 | -0.09 | P01024;Q13361;Q8N584 | 1.36E-02 | -0.02 |
| Q5SQS7 | 9.22E-03 | 3.37 | Q86UW6 | 1.52E-02 | 0.54 |
| P04275 | 9.22E-03 | -0.04 | P10643 | 1.56E-02 | -0.03 |
| Q8NHM5 | 9.22E-03 | -0.04 | Q5S007 | 1.63E-02 | -0.09 |
| P01042 | 9.22E-03 | -0.04 | P07225 | 1.69E-02 | -0.04 |
| O15078;A1L390;P61011;Q96FX2;Q96QE5;Q96T23;Q9BQ75;Q9Y2R4 | 9.22E-03 | -0.09 | Q15772 | 1.84E-02 | 1.18 |
| CE(16:1) Sum Area | 9.22E-03 | -0.04 | Q9UHE8 | 2.31E-02 | -0.51 |
| Q9BTC0 | 9.22E-03 | -0.09 | B4J1X5 | 2.35E-02 | -0.18 |
| A0AVK6 | 9.22E-03 | -0.05 | LPE(18:3) Sum Area | 2.55E-02 | 0.04 |
| Q9NTW7 | 9.22E-03 | 1.69 | P01714 | 2.62E-02 | -0.14 |
| P19827 | 9.22E-03 | -0.03 | Q5JSL3 | 2.62E-02 | -0.07 |
| Cer(d18:1/20:2) Sum Area | 9.75E-03 | -0.03 | Q6ZMZ3 | 2.62E-02 | 0.29 |
| Cer(d18:1/22:2) Sum Area | 9.75E-03 | -0.03 | Q8TAQ2 | 2.72E-02 | 2.53 |
| P08123 | 9.75E-03 | 1.66 | Q969T3 | 2.74E-02 | 1.13 |
| A4FU28 | 9.75E-03 | 2.05 | J9YXX1 | 2.75E-02 | -0.23 |
| Q68DX3 | 9.75E-03 | 0.18 | Q8IYE0 | 2.79E-02 | 0.33 |
| 75B6P5;P01615 | 9.75E-03 | -0.12 | MG(16:0) Sum Area | 3.13E-02 | -0.01 |
| 87WW87;P01614 | 9.75E-03 | -0.06 | Q9P246 | 3.42E-02 | 1.46 |
| Q15848 | 1.12E-02 | -0.13 | Q92542 | 3.42E-02 | -0.13 |
| Q6W0C5 | 1.15E-02 | -2.07 | Q14573 | 3.47E-02 | -0.05 |
| P07996 | 1.15E-02 | -0.09 | P17936 | 3.47E-02 | -0.12 |

**Figure S1**: Ability of machine learning classifiers to identify participants by clinical diagnosis (A) test set AUC showing individual ROC curves, HC and BPH participants (B) test set confusion matrix, HC and BPH participants, with Fisher’s exact test p = 0.16


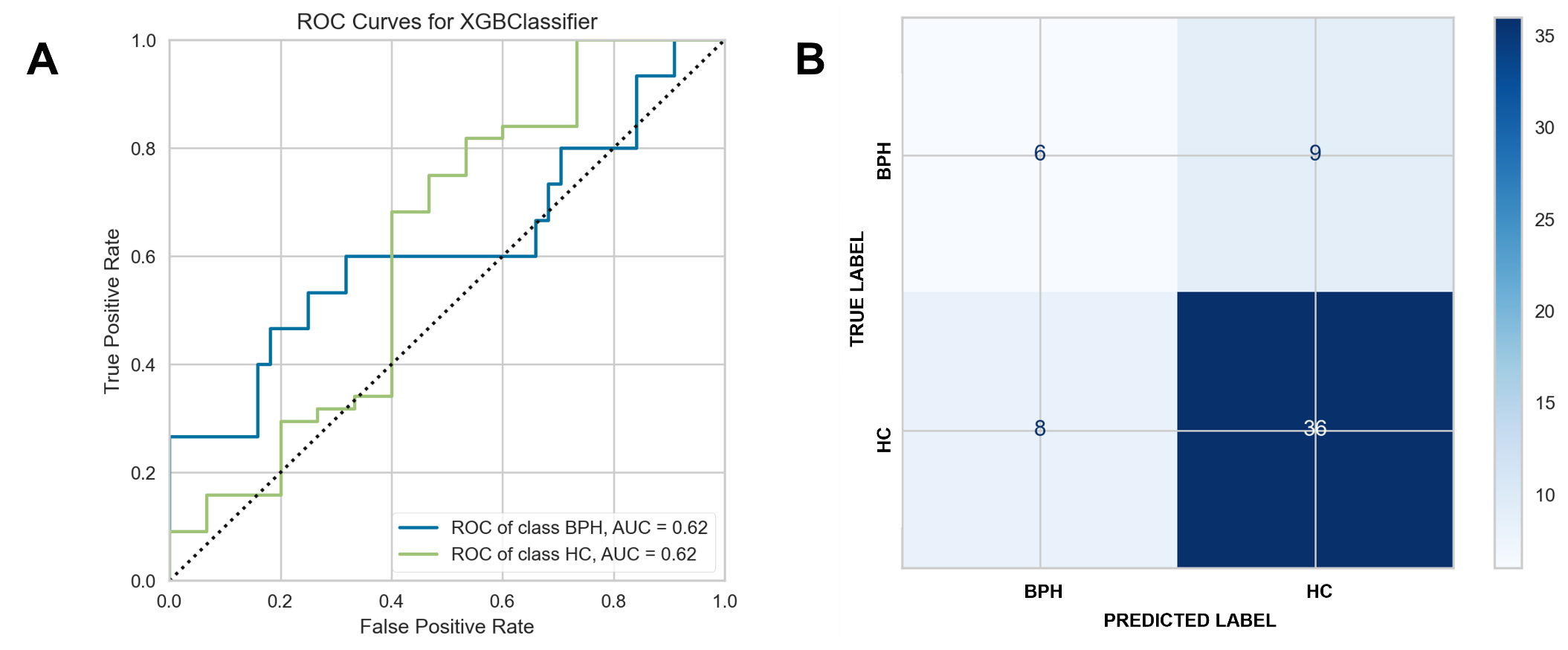


**Table S4**: Pathway analysis using protein biomarkers separating PCa from HC: pathways shown by number of genes identified. Only functions or pathways with 4+ protein biomarkers and statistical significance (p-value <0.05) are shown; p-values are corrected by Bonferroni step-down.

| Term | Group PValue Corrected with Bonferroni step down | Associated Genes Found |
| --- | --- | --- |
| Regulation of Complement cascade | 4.99E-20 | [C2, C3, C4A, C4B_2, C5, C6, C7, C8A, C8B, C9, CFH, CFHR1, CFHR2, CFHR3, CFHR4, CFI, CFP, CPB2, CPN2, F2, PROS1, VTN] |
| Activation of C3 and C5 | 4.99E-20 | [C2, C3, C4A, C4B_2, C5, CFP] |
| Initial triggering of complement | 4.99E-20 | [C2, C3, C4A, C4B_2, CFD, CFP, CRP, FCN2, FCN3, MBL2] |
| Complement cascade | 4.99E-20 | [C2, C3, C4A, C4B_2, C5, C6, C7, C8A, C8B, C9, CFD, CFH, CFHR1, CFHR2, CFHR3, CFHR4, CFI, CFP, CPB2, CPN2, CRP, F2, FCN2, FCN3, MBL2, PROS1, VTN] |
| Response to elevated platelet cytosolic Ca2+ | 4.00E-13 | [A1BG, A2M, ALB, APOA1, CFD, CLEC3B, F13A1, FGA, FGB, FGG, FN1, HRG, IGF2, ITIH3, ITIH4, KNG1, LGALS3BP, ORM2, PECAM1, PF4, PFN1, PLG, PROS1, SELENOP, SERPINA1, SERPINA3, SERPINA4, SERPINF2, TAGLN2, TF, THBS1, VCL, VWF] |
| Platelet activation, signaling and aggregation | 4.00E-13 | [A1BG, A2M, ALB, APOA1, CFD, CLEC3B, COL1A2, DGKZ, F13A1, F2, FGA, FGB, FGG, FN1, HRG, IGF2, ITIH3, ITIH4, ITPR2, ITPR3, KNG1, LGALS3BP, MAPK1, MAPK14, ORM2, PECAM1, PF4, PFN1, PIK3CA, PIK3R6, PLG, PROS1, SELENOP, SERPINA1, SERPINA3, SERPINA4, SERPINF2, TAGLN2, TF, THBS1, VCL, VWF] |
| Platelet degranulation | 4.00E-13 | [A1BG, A2M, ALB, APOA1, CFD, CLEC3B, F13A1, FGA, FGB, FGG, FN1, HRG, IGF2, ITIH3, ITIH4, KNG1, LGALS3BP, ORM2, PECAM1, PF4, PFN1, PLG, PROS1, SELENOP, SERPINA1, SERPINA3, SERPINA4, SERPINF2, TAGLN2, TF, THBS1, VCL, VWF] |
| Hemostasis | 4.00E-13 | [A1BG, A2M, ACTB, ALB, APOA1, CENPE, CFD, CLEC3B, COL1A2, DGKZ, DOCK1, DOCK10, DOCK11, DOCK4, DOCK7, DOCK9, F13A1, F2, F9, FGA, FGB, FGG, FN1, HBD, HBG1, HBG2, HRG, IGF2, IGLL1, ITIH3, ITIH4, ITPR2, ITPR3, JCHAIN, KIF11, KIF13B, KIF16B, KIF1B, KIF21B, KIF27, KIF3B, KIF4A, KLKB1, KNG1, LGALS3BP, MAPK1, MAPK14, ORM2, PDE11A, PECAM1, PF4, PF4V1, PFN1, PIK3CA, PIK3R6, PLG, PROC, PROS1, RAB5A, SELENOP, SERPINA1, SERPINA3, SERPINA4, SERPINA5, SERPINC1, SERPIND1, SERPINF2, SH2B1, SH2B2, TAGLN2, TF, THBS1, VCL, VWF] |
| Formation of Fibrin Clot (Clotting Cascade) | 3.82E-12 | [A2M, F13A1, F2, F9, FGA, FGB, FGG, KLKB1, KNG1, PF4, PF4V1, PROC, PROS1, SERPINA5, SERPINC1, SERPIND1, VWF] |
| Common Pathway of Fibrin Clot Formation | 3.82E-12 | [F13A1, F2, FGA, FGB, FGG, PF4, PF4V1, PROC, PROS1, SERPINA5, SERPINC1, SERPIND1] |
| Intrinsic Pathway of Fibrin Clot Formation | 3.82E-12 | [A2M, F2, F9, KLKB1, KNG1, PROC, PROS1, SERPINA5, SERPINC1, SERPIND1, VWF] |
| Post-translational protein phosphorylation | 1.44E-10 | [ALB, APOA1, APOA2, APOE, APOL1, C3, C4A, FGA, FGG, FN1, FSTL1, IGFBP3, ITIH2, KNG1, MELTF, P4HB, PROC, SCG2, SERPINA1, SERPINC1, SERPIND1, TF, VCAN] |
| Regulation of Insulin-like Growth Factor (IGF) transport and uptake by Insulin-like Growth Factor Binding Proteins (IGFBPs) | 1.44E-10 | [ALB, APOA1, APOA2, APOE, APOL1, C3, C4A, F2, FGA, FGG, FN1, FSTL1, IGF2, IGFALS, IGFBP3, ITIH2, KNG1, MELTF, P4HB, PLG, PROC, SCG2, SERPINA1, SERPINC1, SERPIND1, TF, VCAN] |
| HDL remodeling | 1.92E-06 | [ALB, APOA1, APOC2, APOE, LCAT, PLTP] |
| Chylomicron remodeling | 1.92E-06 | [APOA1, APOA2, APOA4, APOC2, APOE] |
| Plasma lipoprotein remodeling | 1.92E-06 | [ALB, APOA1, APOA2, APOA4, APOC2, APOE, APOF, LCAT, MTTP, P4HB, PLTP] |
| Plasma lipoprotein assembly | 1.92E-06 | [A2M, APOA1, APOA2, APOA4, APOC1, APOC2, APOC4, APOE, MTTP, P4HB] |
| Chylomicron assembly | 1.92E-06 | [APOA1, APOA2, APOA4, APOC2, APOE, MTTP, P4HB] |
| VLDL assembly | 1.92E-06 | [APOC1, APOC4, MTTP, P4HB] |
| Plasma lipoprotein assembly, remodeling, and clearance | 1.92E-06 | [A2M, ALB, APOA1, APOA2, APOA4, APOC1, APOC2, APOC4, APOE, APOF, CLTC, LCAT, MTTP, P4HB, PLTP, SOAT1] |
| Terminal pathway of complement | 2.05E-06 | [C5, C6, C7, C8A, C8B, C9] |
| Innate Immune System | 1.21E-05 | [A1BG, ACTB, ACTG1, ACTR1B, ARHGAP9, ATP8B4, BST1, C2, C3, C4A, C4B_2, C5, C6, C7, C8A, C8B, C9, CD14, CEP290, CFD, CFH, CFHR1, CFHR2, CFHR3, CFHR4, CFI, CFP, COMMD9, CPB2, CPN2, CRP, CYFIP1, DEFA1B, DEFA3, DOCK1, DSP, ELMO2, EPPIN-WFDC6, F2, FCN2, FCN3, FGA, FGB, FGG, GSN, HP, HSP90AA1, HUWE1, IGF2R, IKBKE, ITPR2, ITPR3, KRT1, LBP, LRRFIP1, LTF, LYZ, MAPK1, MAPK14, MAPK8, MBL2, MYO5A, NCSTN, ORM2, PADI2, PECAM1, PGLYRP2, PIK3CA, PROS1, PRSS3, PSMC6, RAB18, RAB31, RAB5B, RNASE3, S100A8, SERPINA1, SERPINA3, STBD1, TTR, VCL, VTN, XRCC5] |
| Binding and Uptake of Ligands by Scavenger Receptors | 1.39E-05 | [ALB, AMBP, APOA1, APOE, APOL1, CD163, COL1A2, HP, HPX, HSP90AA1, JCHAIN] |
| Scavenging of heme from plasma | 1.39E-05 | [ALB, AMBP, APOA1, APOL1, CD163, HP, HPX, JCHAIN] |
| Factors involved in megakaryocyte development and platelet production | 5.33E-05 | [ACTB, CENPE, DOCK1, DOCK10, DOCK11, DOCK4, DOCK7, DOCK9, HBD, HBG1, HBG2, KIF11, KIF13B, KIF16B, KIF1B, KIF21B, KIF27, KIF3B, KIF4A, RAB5A, SH2B1, SH2B2] |
| Signaling by RAF1 mutants | 0.000144 | [ACTB, ACTG1, FGA, FGB, FGG, FN1, MAPK1, VCL, VWF] |
| Signaling downstream of RAS mutants | 0.000144 | [ACTB, ACTG1, FGA, FGB, FGG, FN1, MAPK1, PEBP1, VCL, VWF] |
| Paradoxical activation of RAF signaling by kinase inactive BRAF | 0.000144 | [ACTB, ACTG1, FGA, FGB, FGG, FN1, MAPK1, PEBP1, VCL, VWF] |
| Signaling by BRAF and RAF1 fusions | 0.000144 | [ACTB, ACTG1, AKAP9, FGA, FGB, FGG, FN1, MAPK1, MPRIP, PEBP1, VCL, VWF] |
| Signaling by RAS mutants | 0.000144 | [ACTB, ACTG1, FGA, FGB, FGG, FN1, MAPK1, PEBP1, VCL, VWF] |
| Signaling by high-kinase activity BRAF mutants | 0.000144 | [ACTB, ACTG1, FGA, FGB, FGG, FN1, MAPK1, PEBP1, VCL, VWF] |
| Signaling by moderate kinase activity BRAF mutants | 0.000144 | [ACTB, ACTG1, FGA, FGB, FGG, FN1, MAPK1, PEBP1, VCL, VWF] |
| MAP2K and MAPK activation | 0.000144 | [ACTB, ACTG1, FGA, FGB, FGG, FN1, MAPK1, PEBP1, VCL, VWF] |
| NR1H3 & NR1H2 regulate gene expression linked to cholesterol transport and efflux | 0.000192 | [APOC1, APOC2, APOC4, APOD, APOE, KDM3A, NCOR2, PLTP, RXRA, RXRB] |
| NR1H2 and NR1H3-mediated signaling | 0.000192 | [APOC1, APOC2, APOC4, APOD, APOE, KDM3A, NCOR2, PLTP, RXRA, RXRB] |
| RAC1 GTPase cycle | 0.000236 | [AMIGO2, ARAP2, ARHGAP23, ARHGAP35, ARHGAP9, ARHGEF4, CDC42EP4, CIT, CYFIP1, DIAPH3, DOCK1, DOCK10, DOCK11, DOCK4, DOCK7, DOCK9, FAM13A, PIK3CA, PLEKHG3, PREX1, TAOK3] |
| RHO GTPase cycle | 0.000236 | [ACTB, ACTG1, AKAP13, AMIGO2, ANKRD26, ARAP2, ARHGAP23, ARHGAP35, ARHGAP9, ARHGEF4, CCDC88A, CCT7, CDC42EP4, CIT, CLTC, CYFIP1, DIAPH3, DOCK1, DOCK10, DOCK11, DOCK4, DOCK7, DOCK9, DSP, ELMO2, FAM13A, FGD4, GJA1, HSP90AA1, LETM1, MYO9A, PIK3CA, PLEKHG3, PLXNB1, POTEE, PREX1, RHOV, RNF20, STBD1, TAOK3] |
| Formation of annular gap junctions | 0.00026 | [ACTB, ACTG1, CLTC, CLTCL1, GJA1] |
| Gap junction degradation | 0.00026 | [ACTB, ACTG1, CLTC, CLTCL1, GJA1] |

**Table S5**: Pathway analysis using protein biomarkers separating BPH from HC: pathways shown by number of genes identified. Only functions or pathways with 4+ protein biomarkers and statistical significance (p-value <0.05) are shown; p-values are corrected by Bonferroni step-down..

| Term | Group PValue Corrected with Bonferroni step down | Associated Genes Found |
| --- | --- | --- |
| Hemostasis | 2.09E-06 | [A1BG, A2M, ALB, APOH, COL1A2, DOCK1, DOCK10, DOCK11, DOCK4, DOCK7, HBG1, HBG2, HRG, ITIH3, ITIH4, ITPR3, KIF1B, KIF21B, KIF4A, KLKB1, KNG1, MAPK14, PIK3R6, PLG, PROS1, SERPIND1, SH2B1, TF, THBS1, VWF] |
| Factors involved in megakaryocyte development and platelet production | 1.88E-04 | [DOCK1, DOCK10, DOCK11, DOCK4, DOCK7, HBG1, HBG2, KIF1B, KIF21B, KIF4A, SH2B1] |
| Intrinsic Pathway of Fibrin Clot Formation | 1.12E-04 | [A2M, KLKB1, KNG1, PROS1, SERPIND1, VWF] |
| Formation of Fibrin Clot (Clotting Cascade) | 1.12E-04 | [A2M, KLKB1, KNG1, PROS1, SERPIND1, VWF] |
| Regulation of Insulin-like Growth Factor (IGF) transport and uptake by Insulin-like Growth Factor Binding Proteins (IGFBPs) | 3.74E-05 | [ALB, APOA2, C3, IGFBP3, ITIH2, KNG1, MELTF, P4HB, PLG, SERPIND1, TF] |
| Post-translational protein phosphorylation | 3.74E-05 | [ALB, APOA2, C3, IGFBP3, ITIH2, KNG1, MELTF, P4HB, SERPIND1, TF] |
| Complement cascade | 1.70E-09 | [C3, C7, C8A, CFH, CFHR1, CFHR2, CFI, CPB2, CPN2, FCN3, PROS1, VTN] |
| Regulation of Complement cascade | 1.70E-09 | [C3, C7, C8A, CFH, CFHR1, CFHR2, CFI, CPB2, CPN2, PROS1, VTN] |
| Platelet degranulation | 1.58E-05 | [A1BG, A2M, ALB, APOH, HRG, ITIH3, ITIH4, KNG1, PLG, PROS1, TF, THBS1, VWF] |
| Platelet activation, signaling and aggregation | 1.58E-05 | [A1BG, A2M, ALB, APOH, COL1A2, HRG, ITIH3, ITIH4, ITPR3, KNG1, MAPK14, PIK3R6, PLG, PROS1, TF, THBS1, VWF] |
| Response to elevated platelet cytosolic Ca2+ | 1.58E-05 | [A1BG, A2M, ALB, APOH, HRG, ITIH3, ITIH4, KNG1, PLG, PROS1, TF, THBS1, VWF] |
